# Challenges in the Management of Giant Strangulated Hiatal Hernia: A Case Report

**DOI:** 10.1155/cris/3464069

**Published:** 2026-11-07

**Authors:** Abdellah Nouri, Youssef Ghaddou, Ahmed Bensaad, Abdelaziz Fadil, Khalid Sair

**Affiliations:** ^1^ Mohammed VI Faculty of Medicine Mohammed VI University of Sciences and Health, UM6SS Casablanca Morocco um6ss.ma

**Keywords:** diaphragmatic crus, hiatal hernia, laparoscopic surgery, necrosis, strangulated hernia

## Abstract

A giant hiatal hernia (GHH) is generally defined as the herniation of more than half of the stomach into the thoracic cavity, although definitions vary in the literature, with some authors considering herniation of more than one‐third of the stomach sufficient for diagnosis. Strangulation is a rare but life‐threatening complication that may lead to vascular compromise, gastric ischemia, and ultimately necrosis, requiring urgent diagnosis and individualized surgical management. We report the case of a 54‐year‐old man with long‐standing gastroesophageal reflux disease who presented with chest pain and recurrent vomiting. Contrast‐enhanced computed tomography (CT) demonstrated a giant type III hiatal hernia with approximately two‐thirds of the stomach herniated into the thoracic cavity and associated gastric obstruction. Emergency laparoscopy revealed a severely twisted stomach with extensive necrosis of the fundus and greater curvature. Given the size of the hernia, an adequate exposure of the hiatal area was difficult to achieve, necessitating conversion to a left subcostal laparotomy. After dissection of the two diaphragmatic crus, the sac was reduced and excised, allowing complete reduction of the stomach back into the abdominal cavity. The necrotic areas were managed by longitudinal atypical gastrectomy. Given the large hiatal defect, anterior cruroplasty was performed to prevent an excessive angulation of the esophagus. No antireflux system could be implemented as the fundus was fully removed and Staple‐line integrity was confirmed by an intraoperative methylene blue leak test. The postoperative course was uneventful with minimal residual reflux, treated by proton pump inhibitor therapy. We provide this case to demonstrate the treatment of a giant strangulated hernia and challenges stemming from the alternative surgical approach in laparoscopic and robot‐assisted practice.

## 1. Introduction

A giant hiatal hernia (GHH) is generally defined as the herniation of more than half of the stomach into the thoracic cavity, although some authors consider the herniation of more than one‐third of the stomach sufficient for diagnosis [1]. Strangulation is a rare but life‐threatening complication, most commonly affecting type III hiatal hernias, with reported mortality rates of up to 30% [2]. It may result in upper gastrointestinal obstruction, gastric ischemia, and ultimately gastric necrosis, requiring emergency surgical intervention [2]. Clinical presentation is often nonspecific and may include dysphagia, chest pain, early satiety, vomiting, and upper gastrointestinal bleeding [2].

Laparoscopic repair remains the standard surgical approach, particularly in hemodynamically stable patients, allowing hernia reduction, hiatal defect closure, and antireflux barrier reconstruction [2]. Robotic‐assisted surgery has also emerged as a safe, minimally invasive alternative, providing enhanced dexterity and visualization with postoperative outcomes comparable to those of conventional laparoscopy in selected patients [2].

We report the case of a giant strangulated type‐III hiatal hernia complicated by extensive gastric necrosis that required conversion from laparoscopy to open surgery. This case highlights the intraoperative challenges and surgical decision‐making involved in managing this rare surgical emergency.

## 2. Case Presentation

A 54‐year‐old man with a long‐standing history of gastroesophageal reflux disease, treated with proton pump inhibitors (PPIs) and metoclopramide, presented to the emergency department with chest pain and recurrent vomiting.

On admission, the patient was hemodynamically stable, and physical examination revealed no signs of peritoneal irritation. An electrocardiogram, obtained because of chest pain, showed no abnormalities. Contrast‐enhanced computed tomography (CT) demonstrated a giant type III hiatal hernia with approximately two‐thirds of the stomach, including the gastroesophageal junction and fundus, herniated into the thoracic cavity and causing gastric obstruction (Figure 1).

**Figure 1:**
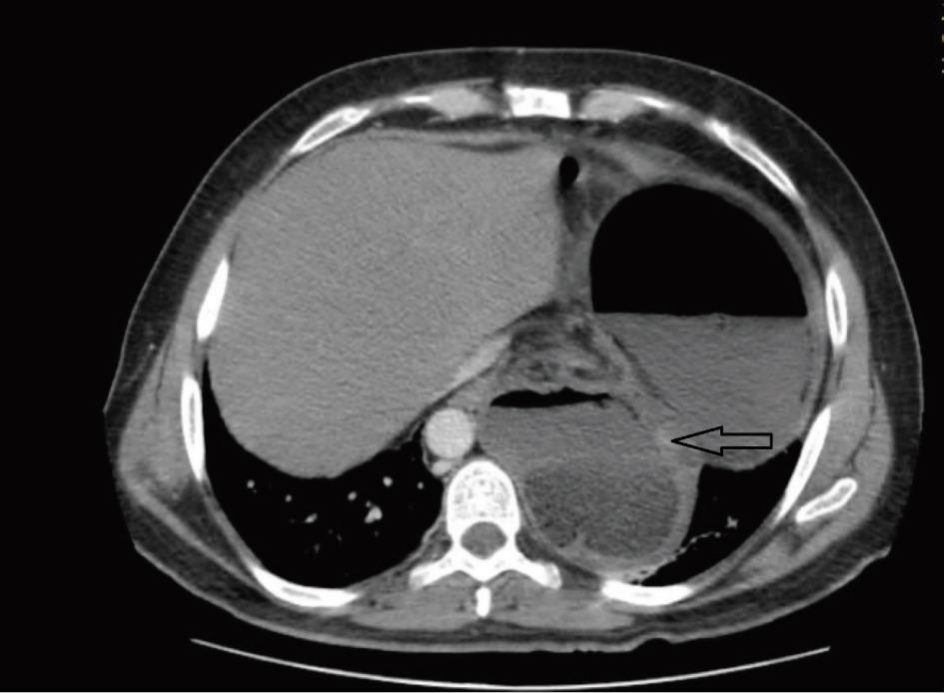
Contrast‐enhanced axial CT image showing a giant type III hiatal hernia with a markedly distended intrathoracic stomach. The arrow indicates the herniated gastric segment; approximately two‐thirds of the stomach was located within the thoracic cavity, producing gastric obstruction.

Laboratory investigations are summarized in Table 1. Based on the clinical presentation and CT findings, a complicated giant type III hiatal hernia with gastric obstruction and suspected volvulus or strangulation was diagnosed. Given the risk of gastric ischemia, the patient was taken urgently to the operating room.

**Table 1:** Patient laboratory results.

Parameter	Patient value	Reference range
Hemoglobin	15.2 g/dL	13–18 g/dL
WBC	17,320 elm/mm^3^	4–11 elm/mm^3^
plt	340,000 elm/mm^3^	150,000–400,000 elm/mm
Na^+^	141 mEq/L	136–145 mEq/L
K^+^	3.2 mEq/L	3.5–5.1 mEq/L
Cl^-^	103 mEq/L	98–107 mEq/L
Créatinine	13.7 mg/L	7–13 mg/L
Alkaline reserve	20 mEq/L	22–30 mEq/L

Initial exploration revealed a severely twisted stomach with extensive necrotic areas involving the fundus and greater curvature. Because of the intrathoracic position of the stomach and suboptimal exposure of the hiatal region, safe laparoscopic dissection could not be achieved. Therefore, strategic conversion to an open approach through a left subcostal laparotomy was decided (Figure 2).

**Figure 2:**
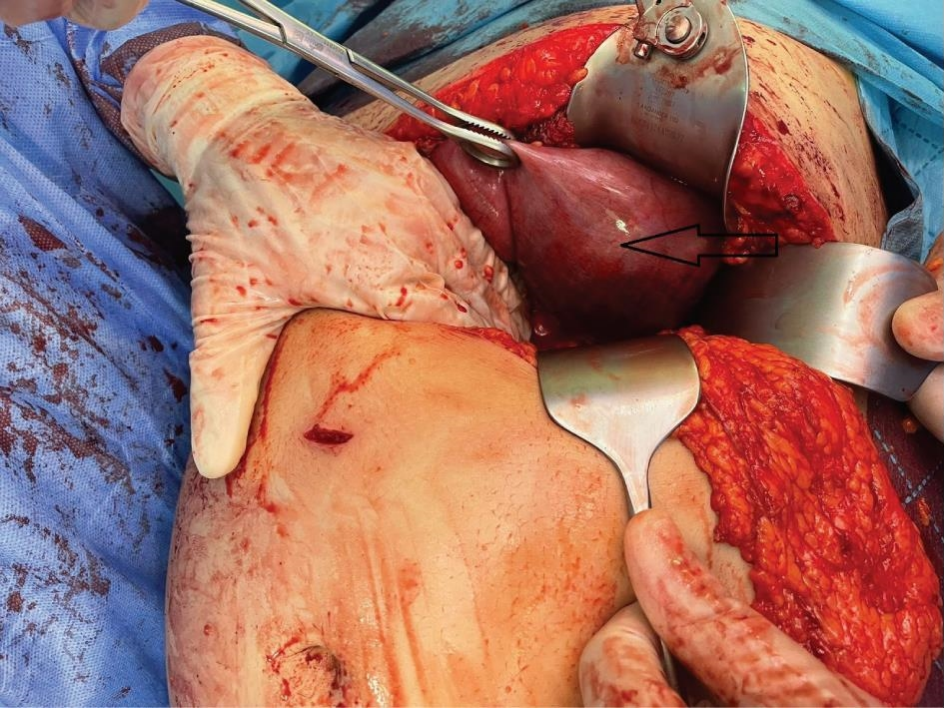
Intraoperative view after conversion to a left subcostal laparotomy. The arrow indicates the reduced stomach, which showed extensive ischemic–necrotic involvement requiring gastric resection.

The pars flaccida was opened, allowing mobilization of the right diaphragmatic crus and access to the posterior epiploic cavity. The short gastric vessels were sealed and divided with a vessel‐sealing device, followed by mobilization of the left diaphragmatic crus. Circumferential dissection of the distal esophagus was completed, enabling reduction and excision of the hernia sac and complete repositioning of the stomach into the abdominal cavity. Gastric viability was assessed macroscopically, as indocyanine green(ICG) fluorescence imaging was not available.

A longitudinal atypical gastrectomy was performed using four 80‐mm GIA stapler reloads, resecting ~60% of the stomach, including the nonviable fundus and greater curvature segment, while preserving a healthy, well‐perfused gastric remnant (Figure 3).

**Figure 3:**
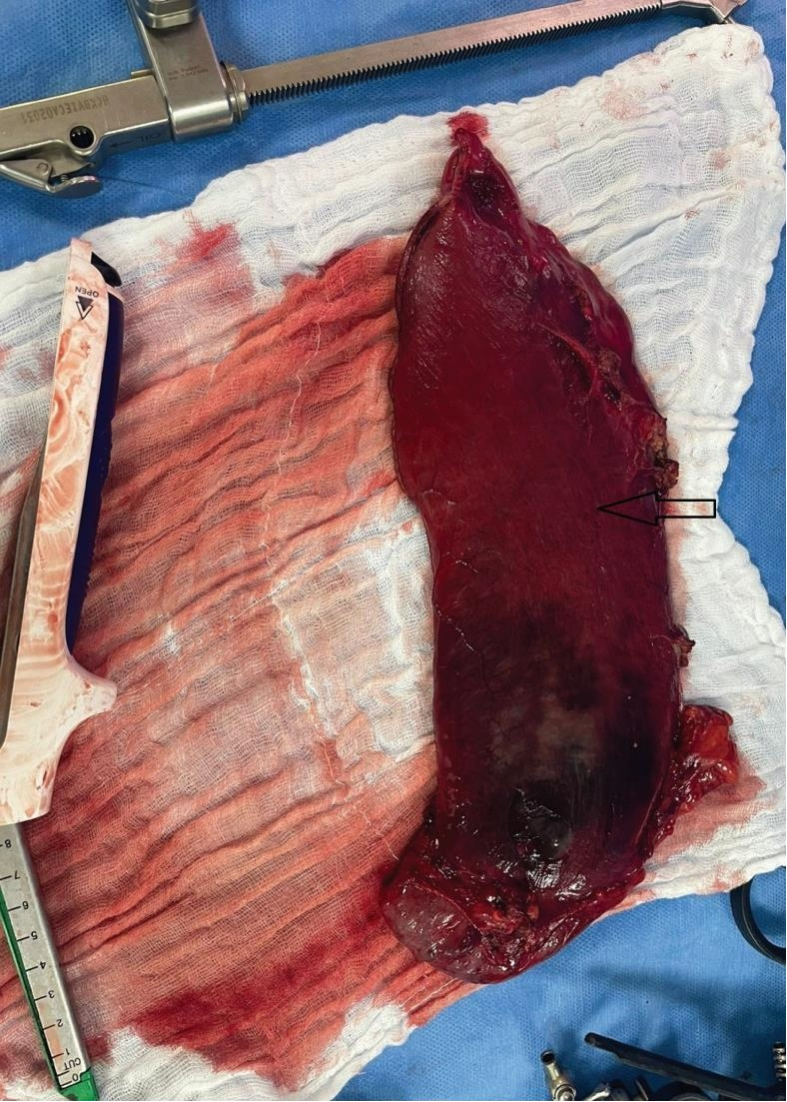
Specimen from the longitudinal atypical gastrectomy. The resection included the necrotic fundus and greater‐curvature segment and represented ~60% of the gastric volume.

The hiatal defect was then repaired by anterior cruroplasty. Given the large and distorted hiatus, posterior approximation was considered likely to create excessive suture tension and anterior displacement of the distal esophagus; anterior closure was therefore selected to preserve an adequate esophageal axis and avoid luminal angulation (Figure 4). Neither fundoplication nor gastropexy was technically feasible because the fundus and a substantial portion of the greater curvature had been resected (Figure 5).

**Figure 4:**
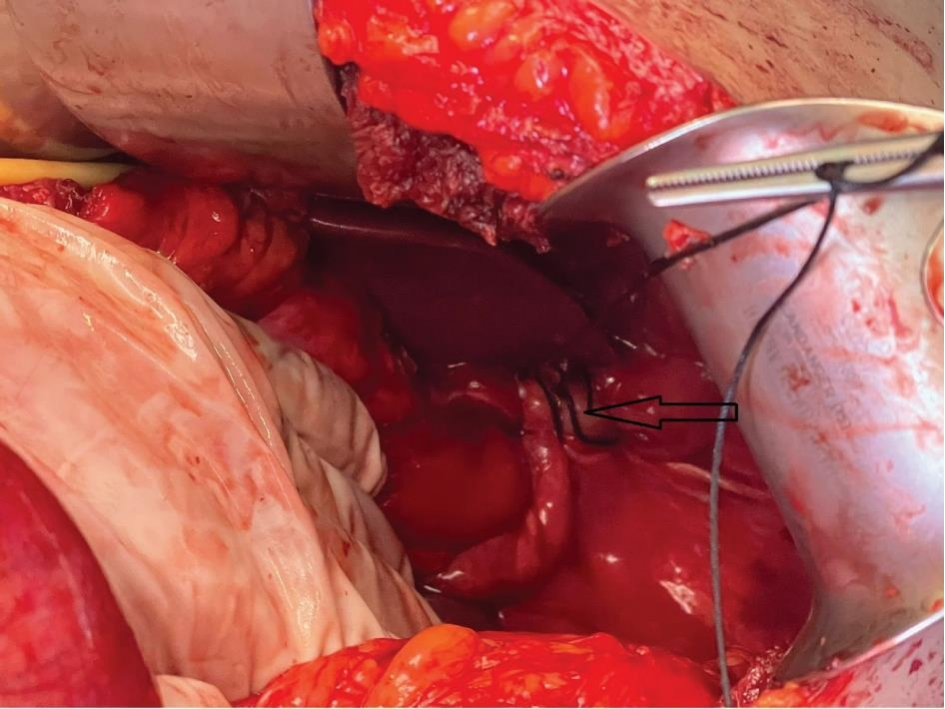
Intraoperative view of anterior cruroplasty. Interrupted sutures approximate the anterior aspect of the diaphragmatic crura while preserving the axis of the distal esophagus and avoiding excessive tension.

**Figure 5:**
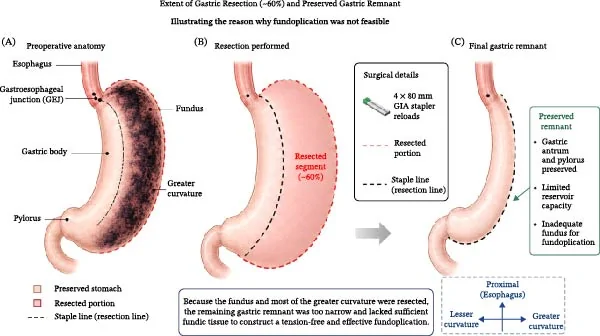
Schematic illustration of the extent of gastric resection. (A) Preoperative distribution of necrosis involving the fundus and greater curvature. (B) Longitudinal resection of the non‐viable segment using four 80‐mm GIA stapler reloads. (C) Final preserved gastric remnant. Loss of viable fundic tissue explains why a tension‐free fundoplication was not feasible.

Staple line integrity was confirmed by an intraoperative methylene blue test administered through the nasogastric tube, with no evidence of leakage. A closed‐suction drain was positioned along the gastric remnant. Operative time was 2 h 40 min, and estimated blood loss was 80 mL.

The patient spent 2 days in the intensive care unit, followed by 3 days in the surgical ward. He remained nil per os for three postoperative days, with a nasogastric tube maintained for decompression. The tube was then removed, and a clear liquid diet was gradually introduced.

The postoperative course was uneventful, with no clinical or biochemical evidence of staple‐line leakage, gastric fistula, or intra‐abdominal sepsis. In our institution, we do not routinely perform postoperative contrast imaging in clinically uncomplicated patients; therefore, oral intake was resumed based on clinical assessment. The abdominal drain was removed on postoperative Day 4, and the patient was discharged on postoperative Day 5.

At a 3‐year follow‐up, the patient remained clinically well, with no symptoms suggestive of recurrent hiatal hernia. Mild residual gastroesophageal reflux was adequately controlled with PPI therapy. Metoclopramide was administered for 4 months postoperatively. Upper gastrointestinal endoscopy performed at 6 months showed no evidence of recurrent hiatal hernia and demonstrated only mild gastritis. No additional radiological or endoscopic assessment was performed thereafter; therefore, the long‐term absence of recurrence was assessed clinically rather than anatomically.

## 3. Discussion

Hiatal hernia is strongly associated with aging and obesity, the latter contributing to increased intra‐abdominal pressure [3]. These two risk factors are also described in patients presenting with acute hiatal hernia complications [2]. Clinical presentation is often nonspecific, with manifestations like simple gastroesophageal reflux and chest pain, while vomiting is more suggestive of gastric strangulation [3]. Our patient had a history of gastroesophageal reflux disease treated with PPI and presented with vomiting and chest pain.

In the elective setting, evaluation of hiatal hernia may include barium swallow, upper gastrointestinal endoscopy, and esophageal manometry [4–7]. In contrast, patients presenting with suspected strangulation require urgent evaluation, making contrast‐enhanced (CT) the imaging modality of choice. CT accurately confirms the diagnosis, demonstrates the extent of gastric herniation, and identifies complications including gastric obstruction, strangulation, pneumomediastinum, and pneumoperitoneum [8, 9]. In our patient, the combination of suggestive clinical findings and CT evidence of a complicated GHH with gastric obstruction raised concern for gastric volvulus or strangulation and prompted immediate surgical intervention without delaying additional investigations, in accordance with the Society of American Gastrointestinal and Endoscopic Surgeons (SAGES) recommendations [10].

Laparoscopic repair is currently the standard for hiatal hernia surgery, with several advantages over laparotomy including less pain, shorter hospital stay, and lower risk of incisional hernia [11]. However, it remains technically challenging in emergency settings with giant hernias complicated by gastric ischemia or necrosis.

Robotic‐assisted surgery has recently emerged as a safe and effective minimally invasive alternative for hiatal hernia repair, offering enhanced three‐dimensional visualization and increased magnification; the articulated instruments offered by robotic systems may facilitate complex hiatal dissections [12–14]. In our institution, however, robotic surgery is currently reserved for elective procedures and was therefore not considered in this emergency case.

Despite these advantages, emergency repair of giant strangulated hiatal hernias remains technically demanding, and conversion to an open approach may be required in selected cases [15]. Conversion to an open procedure should not be regarded as a surgical failure [16] but rather as a deliberate intraoperative strategy when adequate exposure cannot be safely achieved or when gastric viability is compromised [17]. The Delphi consensus by Blikkendaal et al. [18] further distinguishes strategic conversion, performed early when safe laparoscopic completion is considered unlikely, from reactive conversion, which occurs after prolonged unsuccessful dissection or intraoperative complications. This distinction emphasizes that timely conversion represents sound surgical judgment rather than technical failure [18]. In the present case, the combination of a large intrathoracic stomach, limited access to the hiatus, and extensive gastric necrosis precluded safe laparoscopic dissection. A strategic conversion to a left subcostal laparotomy therefore facilitated definitive treatment while avoiding prolonged and potentially hazardous laparoscopic dissection.

Assessing gastric viability is a critical intraoperative challenge in strangulated GHH as it determines the decision to perform gastric resection and its extent. It should be guided by the presence of irreversible ischemia while preserving as much viable stomach as possible to maintain postoperative gastric function [10]. Recent WSES recommendations support the use of ICG fluorescence angiography to assist intraoperative decision‐making in patients with strangulated bowel or gastrointestinal ischemia whenever the technology is available while emphasizing that its implementation in emergency surgery remains limited by equipment availability and institutional resources [19]. Therefore, macroscopic assessment of tissue viability remains fundamental to surgical judgment, particularly in emergency settings where ICG fluorescence imaging may not be available. Obvious transmural gastric necrosis remains a macroscopic diagnosis requiring prompt surgical resection, particularly in emergency settings where ICG may not be available [19]. In our patient, the decision to perform a longitudinal atypical gastrectomy was based on the macroscopic extent of ischemia while aiming to preserve adequate gastric function.

Surgical management begins with careful dissection of both diaphragmatic crura, followed by reduction of the hernia sac and stomach into the abdominal cavity [11, 20]. Extensive mediastinal dissection is recommended to achieve at least 2–3 cm of tension‐free intra‐abdominal esophageal length, which has been associated with a lower risk of recurrence. Although complete excision of the hernia sac is not mandatory, adequate sac dissection is recommended as part of the repair [10]. According to the (SAGES) guidelines, gastric volvulus should be managed by reduction of the stomach, with limited gastric resection when nonviable tissue is encountered [10]. Posterior approximation of the diaphragmatic crura remains the conventional technique for hiatal closure [10, 14, 15]. Although posterior repair is generally recommended, the optimal crural configuration may need to be individualized in patients with large or distorted hiatal defects [21]. In the present case, posterior approximation of the crura was considered likely to create excessive tension and anterior displacement of the distal esophagus, potentially resulting in angulation or luminal narrowing. An anterior cruroplasty was therefore selected as an individualized, expert‐based intraoperative decision adapted to the anatomy of the defect. This approach was intended to achieve hiatal closure while preserving a satisfactory esophageal axis and avoiding excessive tension on the crural sutures. Given the limited published evidence specifically supporting isolated anterior cruroplasty in GHH, this technical choice should not be interpreted as a generally recommended alternative to posterior repair.

The use of mesh for hiatal reinforcement remains controversial. Although mesh reinforcement has been proposed to reduce recurrence after hiatal hernia repair, current SAGES guidelines do not recommend its routine use because of insufficient evidence supporting its long‐term benefit [10]. Absorbable mesh is generally preferred over permanent prosthetic material, which have been associated with serious mesh‐related complications, including erosion and migration [6, 14]. Furthermore, in emergency settings involving gastric ischemia, necrosis, or gastric resection, prosthetic reinforcement is generally avoided because of the potential risk of mesh‐related infection and erosion, making primary suture repair without the mesh consistent with current surgical practice [22, 23]. Accordingly, mesh reinforcement was not used in our patient because of the presence of extensive gastric necrosis and the associated risk of infection.

In most published series, Nissen fundoplication is the preferred technique, and gastropexy is often added to reduce recurrence risk (Table 2) [14, 20]. However, most series summarized in Table 2 predominantly report elective minimally invasive or robot‐assisted repair of giant paraesophageal hernias and are therefore not directly comparable with the present emergency case complicated by gastric strangulation, extensive necrosis, and the need for gastric resection. Table 2 is provided primarily to contextualize the surgical strategies and outcomes reported in the literature. However, following extensive gastric resection, the decision to perform an antireflux procedure must be individualized, as fundoplication may be technically impossible and preservation of a well‐vascularized, tension‐free gastric remnant takes priority over restoration of the antireflux barrier [2, 29]. In our patient, resection of the fundus and greater curvature precluded both fundoplication and gastropexy. Under these circumstances, no antireflux procedure was performed.

**Table 2:** Literature review of giant hiatal hernia repair: postoperative outcomes.

Authors/year	Operative time (min)	Fundoplication (%/type)	Gastropexy	Conversion (%)	Recurrence	Mesh reinforcement (type)
Galvani et al. (2016) [24]	186	93% Nissen; 2% Toupet	No	0	42%	Bio‐A
Sarkaria et al. (2017) [25]	277	63% Nissen	37%	0	NA	Biological
Vasudevan et al. (2017) [26]	83	57% Nissen	Gastrostomy 32%	0	3.6%	Biological (67.9%)
Luna et al. (2020) [27]	146	Nissen	Gastrostomy	20	0%	Parietex
Arcerito et al. (2020) [28]	223	82.9% Nissen; 5.7% Toupet	NA	0	8.6%	Bio‐A (90%)
Ceccarelli et al. (2024) [14]	158	87.2% Nissen; 10.6% Toupet	80%	2.1	4.3%	Bio‐A

Abbreviation: NA, not available.

Recurrence remains the most common long‐term complication following hiatal hernia repair. Multiple contributing factors have been identified, including patient‐related factors, as well as technical factors such as tension‐free repair, adequate intra‐abdominal esophagus length, and, as already debated, the use of mesh may decrease the recurrence risk [30]. Despite the absence of an antireflux procedure in our patient, postoperative reflux remained mild and was adequately controlled with medical therapy during long‐term follow‐up. This case emphasizes that emergency management of strangulated GHH should prioritize safe exposure, complete resection of nonviable tissue, and an individualized reconstruction adapted to the remaining anatomy.

## Funding

No funding was received for this study.

## Consent

Written informed consent was obtained from the patient for the publication of this case report and the accompanying clinical images.

## Conflicts of Interest

The authors declare no conflicts of interest.

## Data Availability

All data supporting the findings of this case report are contained within the article. Additional de‐identified clinical data and source documents relevant to the reported case are available from the corresponding author on reasonable request and will be made available to the journal, editors, or reviewers if required during the review process.
